# Association between Meeting Physical Activity Guidelines and Mortality in Korean Adults: An 8-year Prospective Study

**DOI:** 10.20463/jenb.2016.0054

**Published:** 2017-06-30

**Authors:** Junghoon Kim

**Affiliations:** 1. Department of Preventive Medicine, Gachon University College of Medicine, Incheon Republic of Korea

**Keywords:** Physical activity, Guidelines, Mortality

## Abstract

**[Purpose]:**

Although previous studies have investigated the association between physical activity and various health outcomes, limited information is available on the effect of meeting new governmental guidelines for physical activity on the risk of mortality in Korean adults. This study aimed to examine the prospective association between meeting these guidelines and all-cause mortality during an 8-year follow-up using a large nationwide sample of middle-aged and older adults in Korea.

**[Methods]:**

This prospective study was conducted based on the Korean Longitudinal Study of Ageing. The study participants included 9,177 adults aged 45 years or older at baseline, and all participants were monitored in a 2-year cycle during an 8-year period (70,873 person-years). The risk of mortality was analyzed by determining physical activity levels using the Cox proportional hazard models, and the hazard ratio (HR) and 95% confidence interval (CI) were estimated using Cox models.

**[Results]:**

A statistically significant effect of physical activity on the reduced risk of mortality was observed in cases in which the participants met the guidelines compared to the inactive group (HR: 0.68, 95% CI: 0.58–0.81 vs. the inactive group). A strong association between meeting the recommended physical activity levels and the reduced risk of mortality was also found for non-smokers (HR: 0.62; 95% CI: 0.51–0.76 vs. the inactive group), but not for current smokers (HR: 0.93; 95% CI: 0.67–1.29 vs. the inactive group).

**[Conclusion]:**

Meeting physical activity guidelines was associated with a decreased risk of all-cause mortality for Korean adults. Our results also suggest that smoking reduces the protective effect of physical activity on the risk of mortality.

## INTRODUCTION

Many epidemiological studies have shown that regular physical activity is associated with a range of health benefits, including decreased risk of type 2 diabetes, metabolic syndrome (MetS), cardiovascular disease (CVD), stroke, cancer, and premature mortality, as well as lower medical expenses^1-5^. Other studies have shown that promoting physical activity helps prevent or alleviate chronic diseases such as MetS, type 2 diabetes, and CVD^6-9^. Therefore, developing effective and affordable strategies to encourage physical activity adherence is essential to individuals and society. 

In Korea, the Ministry of Health and Welfare (MHW) compiled current guidelines with recommendations for physical activity for adults in the country^10^. The guidelines indicate that substantial health benefits can be obtained in adults with at least 150 min/week of moderate-intensity activity, at least 75 min/week of vigorous-intensity activity, or an equivalent combination^10^. However, despite the many known health benefits, only 30% of Korean adults meet the recommended levels^11^. Physical inactivity at the population level is also responsible for a substantial health burden in the Korean population and may be associated with increased socioeconomic burden. Previous studies have reported that meeting the minimum levels of physical activity was associated with reduced risk of diabetes, MetS, and CVD^6,7,12^. However, although many studies have reported a positive association between physical activity and reduced risk of chronic diseases, there is limited information on the effect of meeting specific guidelines on the mortality risk. A single study has investigated the decrease in mortality associated with physical activity^4^. Moreover, to the best of our knowledge, no studies have assessed whether meeting current guidelines helps reduce the mortality risk in the Korean population. 

For the above reasons, a population-level strategy intended to promote physical activity and thus reduce the risk of mortality needs to be developed and assessed. Therefore, this study aimed to investigate the effect of meeting physical activity guidelines on the risk of all-cause mortality in the general adult population in Korea using an 8-year nationwide cohort study. 

## METHODS

### Study participants

This prospective cohort study was conducted using data from the Korean Longitudinal Study of Ageing (KLoSA)^13^. KLoSA is an ongoing national prospective study of a representative sample of South Korean adults aged 45 years or older. KLoSA was conducted to produce fundamental data, which were used to establish effective social and economic policies for the Korean aging society. The sampling frame of KLoSA comprises enumeration districts (EDs) clustered in 15 metropolitan cities and provinces, as identified by the National Statistical Office’s 2005 Census. The participants were randomly selected using a multistage and stratified probability sampling design, and the selected household units were stratified by the type of area (urban or rural) and housing (apartment or ordinary [non-apartment]). In 2006, 10,254 participants completed the baseline survey using computer-assisted personal interviewing and were monitored every 2 years until 2014. The study excluded 1,077 (10.5%) respondents with missing data on physical activity, body mass index (BMI), education levels, household income, smoking status, and/or alcohol consumption at baseline. Therefore, 9,177 participants (mean age: 61.0±10.9 years) were eligible and were included in the analysis. All participants signed a written informed consent, and KLoSA was approved by the Institutional Review Board of the Korea Employment Information Service. 

### Assessment of habitual physical activity

All participants self-reported the frequency of physical activity (number of times/week) and duration (minutes). The respondents were asked to provide yes or no answers to whether they participated in physical activity for 1 week, and the frequency (days) and duration (hours) of such activity. The time spent on physical activity was calculated considering the frequency and duration in min/week. The participants were divided into four groups by total physical activity level—none, <75, 75–150, or ≥150 min/week—on the basis of the MHW’s current guidelines on physical activity levels^10^. 

### Mortality

In the present study, the main outcome was the risk of all-cause mortality. This outcome was investigated during follow-up (from 2006 to 2014). All information on the causes and dates of death were obtained from family interviews and death certificates. 

### Covariates

The following covariates were analyzed to assess the effects of potential confounders on mortality: age, sex, education level, household income, physical activity, smoking status, alcohol consumption, obesity, and clinical health conditions. Household income was classified into quartiles of the overall population. The education levels were classified as incomplete high school education, complete high school education, or college level. Alcohol consumption was classified into never, ≤1 time/week (light drinkers), 2–3 times/week (moderate drinkers), and ≥4 times/week (heavy drinkers). BMI was computed from body weight and height (kg/m2) and divided into two groups: normal weight and obese (<25 and ≥25 kg/m2, respectively). The participants reported the following physician-diagnosed clinical health conditions: hypertension, diabetes, CVD, stroke, and/or cancer. 

### Statistical analysis

All statistical analyses were conducted using R software version 3.1.1 (The R Foundation, Vienna, Austria) ^14^. Statistical significance was set at *P*<0.05. The baseline characteristics were indicated as percentages. The physical activity levels were analyzed using a chisquare test. The survival probability curve was plotted using the Kaplan–Meier survival curve according to the physical activity levels. The risk of mortality according to the physical activity levels was estimated using the Cox proportional hazard models, and the hazard ratio (HR) and 95% confidence interval (CI) were calculated using Cox models. Three models (models 1, 2, and 3) were developed to assess the effects of covariates on the association between physical activity and the risk of mortality. Model 1 was adjusted for demographic factors, including age, sex, education level, and household income. Model 2 was further adjusted for smoking status and alcohol consumption. Model 3 was further adjusted for obesity, hypertension, diabetes, CVD, stroke, and any cancer at baseline. Sensitivity analysis was conducted using models stratified by age (≥65 years or <65 years), sex (male or female), obesity (obese or non-obese), and smoking status (non-smoker, never/ ex-smoker, or current smoker). 

## RESULTS

Table 1 shows the participants’ characteristics at baseline. This prospective cohort study included 9,177 Korean adults (44.5% men and 55.5% women) aged ≥45 years (Table 1). Overall, 62.2% were aged 65 years or older; 19.6% were current smokers, 5.9% were heavy alcohol drinkers (≥4 times/week), and 22.7% were obese. Only 26.6% performed physical activity for at least 150 min/week as recommended by current guidelines. In addition, 61.3% did not engage in any physical activity each week. 

**Table 1.** Participants’ characteristics at baseline.

Characteristics		Overall (n=9,177)
Age group (%)	<65 years	5704 (37.8)
	≥65 years	3473 (62.2)
Sex (%)	Male	4088 (44.5)
	Female	5089 (55.5)
Education (%)	≤Middle school	5636 (61.4)
	High school	2513 (27.4)
	≥College	1028 (11.2)
Household income (%)	Low	2338 (25.5)
	Lower-middle	2370 (25.8)
	Upper-middle	2742 (29.9)
	High	1727 (18.8)
Smoking status (%)	Never	6486 (70.7)
	Former smoker	889 (9.7)
	Current smoker	1802 (19.6)
Alcohol consumption (%)	None	5628 (61.3)
	Light	2198 (24.0)
	Moderate	814 (8.9)
	Heavy	537 (5.9)
Physical activity (%)	Inactive	5623 (61.3)
	Low	476 (5.2)
	Moderate	637 (6.9)
	Active	2441 (26.6)
Obesity (%)	No	7090 (77.3)
	Yes	2087 (22.7)
Clinical health condition (%)	Hypertension	2445 (26.6)
	Diabetes	1072 (11.7)
	Cardiovascular disease	428 (4.7)
	Stroke	272 (3.0)
	Cancer	219 (2.4)

Values are number (%).

Table 2 shows the frequency of physical activity. Compared with the inactive group, the members of the active group were significantly older and were likely to be male, non-smokers (never or former), light or moderate drinkers, obese, and had higher education and higher income (*P*<0.001 for all cases).

**Figure 1.**
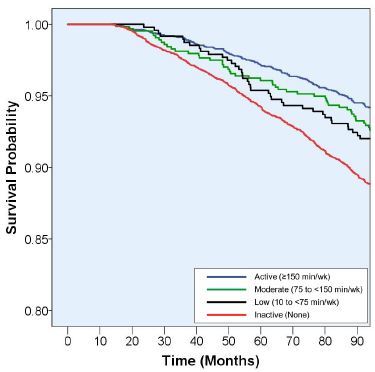
Survival probability according to physical activity levels.

Figure 1 shows the survival probability curve plotted using Kaplan-Meier survival analysis according to the physical activity levels. An inverse association was observed between physical activity levels and mortality (*P*<0.001, Figure 1). Table 3 shows the multivariable HR (95% CI) for the risk of all-cause mortality according to physical activity levels after adjusting for potential confounding factors. Three models were developed to assess the effects of confounders on the association between physical activity and mortality. A significant effect of physical activity on the risk of mortality was observed in model 1 after adjusting for age, sex, education, and household income (*P* for trend <0.001). The association between physical activity and the reduced risk of mortality was strong for the participants who met the guidelines (HR: 0.69; 95% CI: 0.59–0.81). Furthermore, smoking status, and alcohol consumption were controlled in model 2, whereas obesity, hypertension, diabetes, CVD, stroke, and cancer at baseline were controlled in model 3. The significant association between physical activity and the risk of mortality was unchanged in both models. In the fully adjusted model, the HR was 0.68 (95% CI: 0.58–0.81) compared with that of the inactive participants (*P*<0.01). 

**Table 2.** Participants’ characteristics by physical activity levels.

Characteristics		Inactive (n=5,623)	Low (n=476)	Moderate (n=637)	High (n=2,441)	p-value
Age group (%)	<65 years	2375 (42.2)	136 (28.6)	188 (29.5)	774 (31.7)	<*0.001*
	≥65 years	3248 (57.8)	340 (71.4)	449 (70.5)	1667 (68.3)	
Sex (%)	Male	2365 (42.1)	226 (47.5)	307 (48.2)	1190 (48.8)	<*0.001*
	Female	3258 (57.9)	250 (52.5)	330 (51.8)	1251 (51.2)	
Education (%)	≤Middle school	3941 (70.1)	234 (49.2)	308 (48.4)	1153 (47.2)	<*0.001*
	High school	1304 (23.2)	162 (34.0)	222 (34.9)	825 (33.8)	
	≥College	378 (6.7)	80 (16.8)	107 (16.8)	463 (19.0)	
Household income (%)	Low	1561 (27.8)	106 (22.3)	136 (21.4)	535 (21.9)	<*0.001*
	Lower-middle	1588 (28.2)	115 (24.2)	146 (22.9)	521 (21.3)	
	Upper-middle	1635 (29.1)	141 (29.6)	204 (32.0)	762 (31.2)	
	High	839 (14.9)	114 (23.9)	151 (23.7)	623 (25.5)	
Smoking status (%)	Never	3975 (70.7)	346 (72.7)	424 (66.6)	1741 (71.3)	<*0.001*
	Former smoker	437 (7.8)	53 (11.1)	86 (13.5)	313 (12.8)
	Current smoker	1211 (21.5)	77 (16.2)	127 (19.9)	387 (15.9)
Alcohol consumption (%)	None	3588 (63.8)	263 (55.3)	353 (55.4)	1424 (58.3)	<*0.001*
	Light	1235 (22.0)	140 (29.4)	176 (27.6)	647 (26.5)	
	Moderate	455 (8.1)	45 (9.5)	75 (11.8)	239 (9.8)	
	Heavy	345 (6.1)	28 (5.9)	33 (5.2)	131 (5.4)	
Obesity (%)	No	4439 (78.9)	349 (73.3)	497 (78.0)	1805 (73.9)	<*0.001*
	Yes	1184 (21.1)	127 (26.7)	140 (22.0)	636 (26.1)	
Clinical health condition (%)	Hypertension	1486 (26.4)	120 (25.2)	162 (25.4)	677 (27.7)	*0.457*
	Diabetes	618 (11.0)	56 (11.8)	87 (13.7)	311 (12.7)	*0.053*
	Cardiovascular disease	251 (4.5)	21 (4.4)	33 (5.2)	123 (5.0)	*0.631*
	Stroke	168 (3.0)	6 (1.3)	27 (4.2)	71 (2.9)	*0.038*
	Cancer	123 (2.2)	14 (2.9)	13 (2.0)	69 (2.8)	*0.268*

Values are number (%). *P*-values were calculated using Chi-squared test.

**Table 3.** Multivariable hazard ratio (95% CI) for the risk of all-cause mortality according to physical activity levels in Korean adults.

Physical activity levels	Person-years	Deaths/participants	Hazard ratio (95% CI)		
			Model 1	Model 2	Model 3
Inactive	43082	778 / 5623	1.00 (Reference)	1.00 (Reference)	1.00 (Reference)
Low	3699	47 / 476	0.98 (0.73-1.32)	1.02 (0.76-1.38)	1.03 (0.77-1.40)
Moderate	4957	58 / 637	0.99 (0.75-1.30)	0.99 (0.75-1.29)	0.93 (0.71-1.22)
High	19136	178 / 2441	0.69 (0.59-0.81)*	0.70 (0.59-0.82)*	0.68 (0.58-0.81)*
*P for trend*			*<0.001*	*<0.001*	*<0.001*

Values were hazard ratio (95% CI).

Model 1 was adjusted for age, sex, education, and household income.

Model 2 was additionally adjusted for smoking status and alcohol consumption.

Model 3 was additionally adjusted for obesity and clinical health conditions.

CI: confidence interval

*P<0.05 compared with reference group.

**Figure 2.**
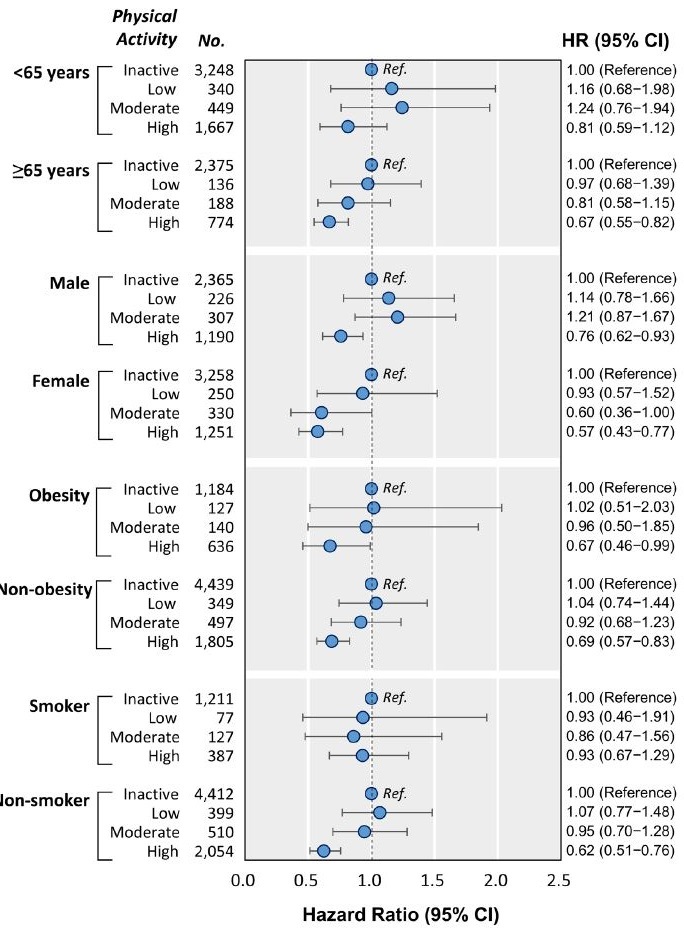
Multivariable hazard ratio (95% confidence interval) for the risk of all-cause mortality according to physical activity levels by subgroups. The values correspond to hazard ratios (95% CI); each model included age, sex, education level, household income, smoking status, alcohol consumption, obesity, hypertension, diabetes, cardiovascular disease, stroke, and cancer.

Sensitivity analysis was conducted using fully adjusted models stratified by age, sex, obesity, and smoking status (Figure 2). The associations were consistent with the results of the overall population, irrespective of sex or the presence/absence of obesity. However, we found a significant protective effect of physical activity on the risk of mortality in the older population (HR: 0.67; 95% CI: 0.55–0.82 for the active group), although this effect was not significant for the middle-aged population. A strong association between meeting the physical activity levels and the reduced risk of mortality was found for non-smokers (HR: 0.62; 95% CI: 0.51–0.76 vs. the inactive group), but not for current smokers (HR: 0.93; 95% CI: 0.67–1.29 vs. the inactive group). 

## DISCUSSION

This large, prospective cohort study aimed to investigate the effect of meeting physical activity guidelines on the risk of all-cause mortality using a nationally representative sample of middle- to older-aged Korean adults. After adjusting for covariates, the results indicated a significant positive correlation with the decreased risk of mortality. Moreover, being a current smoker reduced the protective effects of physical activity on the risk of mortality. 

To the best of our knowledge, this study is the first to examine this association in the Korean population. The new physical activity guidelines for Korea were established using a review of studies conducted in Western countries, and this review summarized evidence on the effects of physical activity on lifestyle-related diseases. The Korean population may differ from that of other countries, including Western countries, for social and urban environments. Therefore, it is important to investigate the effect of meeting the new governmental guidelines for physical activity on the risk of mortality in Korean adults. Our findings indicated that being physically active (≥150 min/week) was associated with a 32% lower risk of mortality compared with physically inactive participants. The anticipated protective effects of physical activity were greater than those of smoking status (HR in never smokers=0.75 vs. current smokers), obesity (HR in non-obese: 0.81 vs. obese), and equivalent to those of age lowered by 4 years (HR=0.68). However, no significant association between low (<75 min/week) and moderate (75 to <150 min/week) levels of physical activity and a reduced risk of mortality was found. Current guidelines for physical activity issued by the World Health Organization and individual countries, including Korea, recommend the execution of physical activity for at least 150 min/week, although individuals can choose their activity patterns according to their lifestyle^15-17^. Therefore, the present findings suggest that a physically active lifestyle that meets the guidelines of at least 150 min/week of activity decreases the risk of mortality and extends longevity in Korean adults. 

To date, only a few epidemiological studies have investigated the effects of meeting physical activity guidelines (≥150 min of moderate activity per week) on the improvement of mortality in a general population. The present study confirmed the findings of these studies on the inverse association between physical activity and mortality risk. Gebel et al. conducted a large population-based prospective study of middle-aged and older Australians and reported that the risk of mortality decreased by 9–13% in cases in which physical activity guidelines were met^18^. Another cohort study reported a lower mortality among women who met such guidelines, although the number of participants was small. A large systematic review with meta-analysis of cohort studies reported that the risk of mortality in the highest physical activity group decreased approximately 29% compared with the study group with the lowest activity^19^. In the present study, the extent of the effect of the protective effect of physical activity on risk of mortality was larger than in comparable studies because participants who any physical activity were used as a reference group. 

Several biological mechanisms have been reported to explain the protective effect of physical activity on decreased risk of mortality. For example, regular physical activity has been reported to improve body composition such as in weight loss and reduction of abdominal adiposity linked to increased high-density lipoprotein cholesterol levels, decreased low-density lipoprotein, and reduced triglyceride levels, as well as improvement of glucose homeostasis and insulin sensitivity^1,20-22^. Physical activity also helps reduce the risk of onset of chronic diseases such as hypertension, type 2 diabetes, and CVD^12,23,24^. Chronic inflammation is also a strong predictor of most chronic diseases, and physical activity offers a preventive benefit^25,26^. A previous study by our research group indicated that physical activity helps improve cardiovascular risk factors in middle-aged individuals with MetS^6^. Moreover, randomized control trial studies have reported that physical activity might significantly reduce C-reactive protein and cardiovascular risk factors^27^. Any or all of these factors may explain the direct or indirect protective effects on the risk of premature death via reduced incidence of chronic disease in populations that engage in regular physical activity. 

The present study also evaluated the modifying impact of physical activity according to age, sex, obesity, and smoking status and found that major predictors affected the lifespan. This study found a significant association between physical activity and the reduced risk of mortality in older populations. Accordingly, previous studies involving older adults provided consistent evidence that physical activity was associated with a reduced risk of mortality. However, the results were not consistent for middle-aged individuals because of the relatively low number of deaths in this group during follow-up. In fact, 77% of all deaths occurred among individuals aged 65 years or older. Therefore, the study period may have been too short to determine the association between mortality rate and physical activity in the middle-aged group. Furthermore, consistent with meta-analyses^19,28^, the present study suggests that the reduced risk of mortality due to physical activity was higher in women than in men. Moreover, a weak association between physical activity and the risk of mortality was observed in the middle-aged population but not in the older population. Gebel et al. reported that physical activity was associated with a decreased risk of mortality in middle-aged adults but not in older adults in a study involving 204,542 Australians^18^. The present study also found a significant association between physical activity and reduced risk of mortality in non-smokers but not in current smokers (P-value for interaction <0.05). Smoking has been found to be a strong predictor of increased risk of many chronic diseases and mortality^29^. Our results revealed a significant increase in the risk of mortality in current smokers compared with never smokers (HR: 1.25 vs. never smokers, data not shown). The findings thus suggest that the negative effects of smoking may weaken the protective effect of physical activity on risk of mortality. 

### Strengths and limitations

The strength of the present study was the use of a large, nationally representative sample, designed prospectively over 8 years, and analyzed after considering potential confounding factors for mortality, including demographics, health-related behaviors, and clinical health conditions. Therefore, the causality between physical activity and the reduced risk of mortality can be inferred, and the findings of the present study can be generalized to the Korean population. 

However, this study has several limitations. First, physical activity was assessed via a self-reported questionnaire, which carries the possibility of bias because it is difficult to determine the intensity of activity accurately using the questionnaire. In fact, the baseline prevalence of meeting physical activity guidelines among our participants (26.6%) was lower than that in the general Korean adult population, as found in the Korea National Health and Nutrition Examination Survey 2007 (31%). Therefore, further studies may be needed to measure intensity-specific physical activity and establish associations with our findings. Second, although confounding factors directly related to the mortality risk were considered in this study, other unexplored, residual confounding factors such as sociocultural factors and genetic variation may partly explain our findings. However, the present findings should help elucidate the association between meeting physical activity guidelines and the decreased risk of mortality and promote public health in the Korean population. 

## CONCLUSIONS

The findings of this prospective study indicate an association between meeting the physical activity guidelines for Korean adults and a decreased risk of mortality. The engagement in physical activity for 150 min per week or longer was also found to decrease the risk of all-cause mortality by 32%. In addition, our results suggest that smoking reduces the protective effects of physical activity on the risk of mortality. 

## COMPETING INTERESTS

The authors declare that they have no competing interests. 
